# Differentiation in Theta and Beta Electrocortical Activity between Visual and Physical Perturbations to Walking and Standing Balance

**DOI:** 10.1523/ENEURO.0207-18.2018

**Published:** 2018-08-13

**Authors:** Steven M. Peterson, Daniel P. Ferris

**Affiliations:** 1 Department of Biomedical Engineering, School of Engineering, University of Michigan, Ann Arbor, MI 48109-2099; 2 J. Crayton Pruitt Family Department of Biomedical Engineering, University of Florida, Gainesville, FL 32611

**Keywords:** balance control, EEG, independent component analysis, perturbation

## Abstract

Human balance is a complex process in healthy adults, requiring precisely timed coordination among sensory information, cognitive processing, and motor control. It has been difficult to quantify brain dynamics during human balance control due to limitations in brain-imaging modalities. The goal of this study was to determine whether by using high-density electroencephalography (EEG) and independent component analysis, we can identify common cortical responses to visual and physical balance perturbations during walking and standing. We studied the responses of 30 healthy young adults to sensorimotor perturbations that challenged their balance. Subjects performed four 10 min trials of beam walking and tandem stance while either being mediolaterally pulled at the waist or viewing brief 20° field-of-view rotations in virtual reality. We recorded high-density EEG, motion capture, lower leg electromyography (EMG), and neck EMG. We hypothesized that both physical pull and visual rotation perturbations would elicit time–frequency fluctuations in theta (4–8 Hz) and beta (13–30 Hz) bands, with increased occipito-parietal activity during visual rotations compared with pull perturbations. Our results confirmed this hypothesis. For both perturbations, we found early theta synchronization and late alpha–beta (8–30 Hz) desynchronization following perturbation onset. This pattern was strongest in occipito-parietal areas during visual perturbations and strongest in sensorimotor areas during pull perturbations. These results suggest a similar time–frequency electrocortical pattern when humans respond to sensorimotor conflict, but with substantive differences in the brain areas involved for visual versus physical perturbations. Our findings may have important implications for assessing and training balance in individuals with and without motor disabilities.

## Visual Abstract

**Figure:**
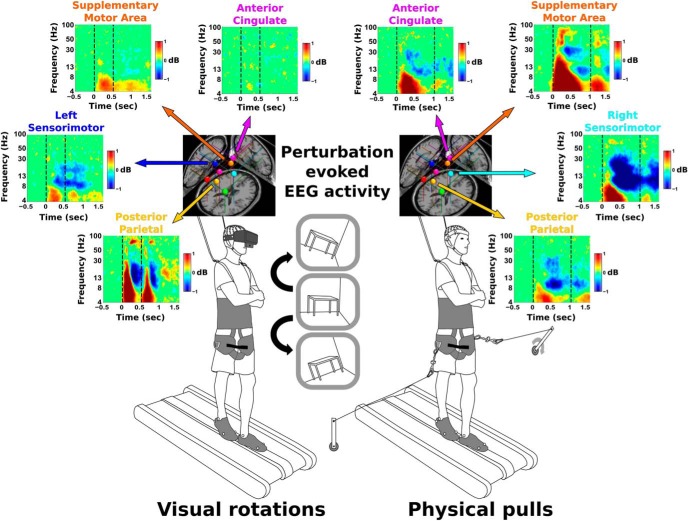

## Significance Statement

We performed the first electromyography (EEG) time–frequency analysis on source-localized human electrocortical responses to physical and visual balance perturbations during both walking and standing. Perturbations elicited similar time–frequency patterns, but in notably different cortical areas for physical versus visual perturbations. Perturbation-evoked EEG fluctuations localized primarily to occipito-parietal areas during visual perturbations and motor areas during physical perturbations. These similarities suggest a common electrocortical response to sensorimotor perturbations. Notably, standing had greater electrocortical responses than walking. The results from this study may have applications in assessing and assisting the treatment of balance dysfunction.

## Introduction

In the real world, humans must constantly make postural adjustments to avoid losing balance. Such adjustments require precise coordination among sensory input, cognitive processing, and motor control (Macpherson and Horak, 2012). Dual-tasking studies have highlighted the importance of human supraspinal centers for maintaining balance during walking and standing (Rankin et al., 2000; Woollacott and Shumway-Cook, 2002). Despite this, our current understanding of real-world human cortical activity in response to balance perturbations is limited (Varghese et al., 2017). Traditional neuroimaging methods, such as functional magnetic resonance imaging and functional near-infrared spectroscopy (fNIRS), are limited by stationary subjects and low temporal resolution.

High-density, source-localized electroencephalography (EEG) is currently the most promising method to noninvasively assess human cognitive activity during balance. The strengths of EEG are its portability and high temporal resolution (Gramann et al., 2011, 2014). High temporal resolution is essential for quantifying brief cortical balance responses. EEG is typically limited by its low spatial resolution and susceptibility to artifact contamination (Urigüen and Garcia-Zapirain, 2015). However, blind-source separation techniques such as independent component analysis can separate out cortical activity from artifacts, both reducing the effects of artifacts and enhancing spatial resolution (Makeig et al., 1996; Gwin et al., 2010). Independent component analysis also allows researchers to draw stronger conclusions about specific brain regions compared with channel data, which contain activity from multiple regions due to volume conduction.

Healthy adult EEG balance studies have focused on theta (4–8 Hz) and beta (13–30 Hz) frequency bands (Varghese et al., 2017). EEG recordings show decreased electrocortical beta power associated with active gait control (Wagner et al., 2014) and more challenging balance tasks (Sipp et al., 2013). Beta power in parietal and central cortical regions has been shown to decrease following sudden changes in gait patterns, indicating that beta power in these areas is involved in motor inhibition (Wagner et al., 2016). In addition, brief EEG theta oscillations occur when subjects lose their balance (Sipp et al., 2013) or are exposed to external perturbations (Varghese et al., 2014). Other healthy adult EEG studies have indicated that theta power may be related to changes in balance performance (Slobounov et al., 2013; Hülsdünker et al., 2015). It seems likely that increased theta and decreased beta power are involved during active balance control and may fluctuate as balance difficulty changes.

In addition to physical manipulations such as pushing the subject or suddenly translating the support surface (Duckrow et al., 1999; Adkin et al., 2008), manipulated sensory information can provide insight into cortical sensory integration during balance control. In contrast to physical perturbations, sensory manipulations such as restricted vision, altered surface firmness, and auditory feedback target specific sensory input (Pirini et al., 2011; Tse et al., 2013). Sensory perturbations are advantageous for EEG experiments because they do not directly move the subject in a consistent manner, unlike physical perturbations, reducing the effects of motion artifact (Kline et al., 2015).

In particular, visual manipulations can greatly impact healthy adult electrocortical dynamics and balance control by inducing conflict among visual, vestibular, and proprioceptive inputs. Blindfolded walking in healthy adults has shown increased EEG spectral power in somatosensory areas (Oliveira et al., 2017b), indicating that visual manipulations can substantially alter electrocortical dynamics. Similarly, visual rotations using prism goggles can increase mediolateral sway during stance due to sensory conflict caused by inaccurate visual input (Cauquil et al., 1998). Also, perturbed optical flow can increase healthy adult parietal theta power (Slobounov et al., 2013). Understanding visual processing during balance control is important because over-reliance on vision is a cause of increased falls in older adults (Franz et al., 2015).

The purpose of our study was to identify similarities and differences in healthy adult electrocortical activity between physical pull and visual rotation perturbations during beam walking and tandem stance. We used a mediolateral pull to the subject’s waist to physically challenge balance. For the visual perturbation, subjects wore a virtual reality head-mounted display that induced a 20° visual field rotation during beam walking and tandem stance. We hypothesized that both the physical pull and visual perturbations would transiently increase theta power (4–8 Hz) and decrease beta power (13–30 Hz), indicating cortical detection of perturbed balance and decreased motor inhibition, respectively. We also expected that visual rotations would increase fluctuations in occipito-parietal areas based on the prominent cortical areas for visual processing whereas physical pull perturbations would increase cortical activity in central sensorimotor areas due to the large EEG event-related potentials seen in these areas during physical perturbations, likely indicating planning of a motor response (Marlin et al., 2014).

## Materials and Methods

### Subjects

We tested 30 healthy, young adults (15 females, 15 males; mean ± SD age, 22.5 ± 4.8 years) for this study. All subjects identified themselves as right hand and right foot dominant, with normal or corrected vision. We screened subjects for any neurologic, orthopedic, or cardiac conditions and injuries. All subjects provided written informed consent. Our protocol was approved by the University of Michigan Health Sciences and Behavioral Sciences Institutional Review Board for the protection of human subjects.

Before each experiment, we screened subjects for motion sickness in virtual reality. Subjects stood in place for a 5 min activity while wearing a virtual reality headset (Oculus Rift DK2, Oculus VR). Subjects walked and jumped around a virtual environment using body gestures tracked by a Microsoft Kinect version 2 (Microsoft). We included a disconnect between real and virtual movements to be more disorienting than our main testing protocol. Subjects participated in the main experiment if both the subject and experimenter agreed that they did not exhibit any symptoms of motion sickness. Two potential subjects exhibited motion sickness symptoms and did not participate in the experiment; 30 subjects passed this screening.

### Experiment design

Subjects either walked at 0.22 m/s or stood on a balance beam that was 2.5 cm in height by 12.7 cm in width mounted to a treadmill. Subjects wore a body-support harness for safety, with extended support straps to allow for unrestricted mediolateral movement. The beam was designed to be wide enough for a single foot to enforce tandem gait and tandem stance. We gave subjects specific instructions to look straight ahead and to avoid looking at their feet. We instructed subjects to move only their hips side-to-side to balance when on the balance beam, avoiding rotations across the longitudinal axis of their body. Subjects also crossed their arms and walked heel-to-toe during the walking conditions. We had subjects cross their arms while walking so that subjects avoided swinging their arms to stabilize themselves. It made the task more difficult and also conformed with previous studies on treadmill balance beam walking (Domingo and Ferris, 2009, 2010; Sipp et al., 2013). Crossed arms can also reduce intersubject variability during the task because there is no variation in arm movement.

**Figure 1.**
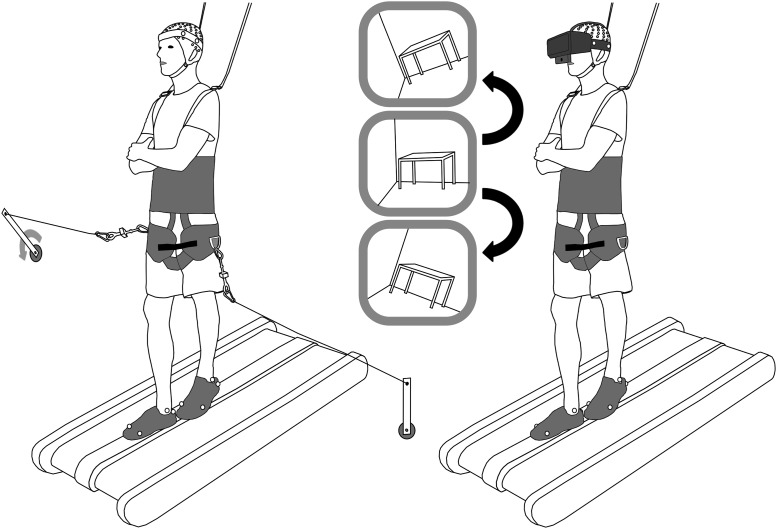
A sketch of a subject walking on the beam, exposed to pull (left) and visual rotation (right) perturbations. Subjects wore a body support harness for all conditions. The inset sketches show example 20° perturbations in counterclockwise (top) and clockwise (bottom) directions. Subjects were exposed to pull and visual rotation perturbations during separate 10 min trials of standing and walking, leading to four total trials per subject.

We used two types of perturbations during testing: a visual field rotation and a mediolateral pull to one side (Fig. 1). We randomly selected half of the subjects to perform visual rotations first while the other half performed the pull perturbations first. The visual field rotation was presented with an Oculus Rift virtual reality headset. Subjects saw the view of a webcam mounted to the headset. This view was digitally rotated 20° clockwise or counterclockwise for 0.5 s. For the pull perturbation, subjects were physically pulled by one of two electromechanical motors (Chiaphua Industries Motor) placed on either side of the treadmill. When commanded (dSPACE), one motor rotated an attached bar 90° away from the subject, which pulled on a steel cable connected to the subject’s safety harness. The motor rotated back 1 s after the initial rotation. Tensile load cells (catalog #LCM703, OMEGA Engineering) were attached in series with the steel cable on either side to record pull force and onset. Both perturbation types were presented using predefined pseudo-random sequences. Subjects were exposed to each perturbation for 10 min conditions of 150 perturbations each (75 per side). We had subjects exposed to each of these perturbations while standing and while walking (four trials total). Subjects were asked to stand in tandem stance, with their right foot in front of the left. Subjects walked at a speed of 0.22 m/s to enable subjects to maintain balance consistently on the balance beam.

We recorded EEG using a 136-channel system (BioSemi Active II, BioSemi), sampled at 512 Hz. Two of the EEG electrodes were used to record left and right neck muscle activity. EEG electrode positions were measured using an ELPOS Digitizer (Zebris Medical). We also recorded motion capture from 16 reflective markers on the feet, sacrum, neck, and head (Vicon). We recorded EMG from 4 lower leg muscles of each leg (tibialis anterior, soleus, medial gastrocnemius, and peroneus longus), sampled at 1000 Hz (Biometrics). We used a 0.5 Hz square pulse to synchronize the recording systems.

To analyze perturbation-evoked activity, we needed to determine perturbation onset events. For visual perturbations, we programmed keyboard button presses on the virtual reality computer keyboard to correspond with perturbation onset, using Windows Input Simulator. This keyboard input was recorded and synchronized with the EEG data using Lab Streaming Layer (Delorme et al., 2011). Pull perturbation events were determined by finding peaks in detrended load cell data. We estimated the pull onset events by finding when the load cell voltage first went 3 SDs above baseline voltage before each peak and manually inspected each to ensure accuracy. We used these onset times as the final pull perturbation events.

### Behavioral and physiologic measures

We used cleaned, mediolateral motion capture marker trajectories from the head and sacrum to estimate perturbation-evoked changes in stability and overall stability during each trial. We estimated body and head sway during each trial with mediolateral SD of the sacrum and head markers. We ran a 2 × 2 repeated-measures ANOVA to test for the main effects of perturbation type (pull vs rotate) and physical task (standing vs walking). *Post hoc* pairwise comparisons were performed using *t* tests with false discovery rate correction (*p* < 0.05; Benjamini and Yekutieli, 2001). We also analyzed the perturbation-evoked head and sacrum mediolateral displacement. Marker trajectories were detrended, 6 Hz low-pass filtered, and fully rectified. We epoched the result around each perturbation onset, subtracted baseline motion for the half-second before perturbation onset, and averaged the result for each perturbation type.

Peak load cell voltages were used to determine whether pull forces differed due to the physical task (standing vs walking) or pull direction (left vs right). We converted peak detrended load cell voltages to pull forces (in newtons) based on prior calibration of the load cells with known weights. A 2 × 2 repeated-measures ANOVA analyzed main effects of physical task and pull direction on peak pull force.

We also analyzed perturbation-evoked EMG activity in the lower leg. EMG data were detrended, 20 Hz high-pass filtered, and full-wave rectified (Sipp et al., 2013). We used 3 min of 0.22 m/s baseline tandem walking to normalize the EMG activity for each muscle electrode. Baseline walking occurred without perturbations and without the virtual reality headset. EMG activity during baseline walking was detrended, 20 Hz high-pass filtered, and full-wave rectified. We then time warped the baseline EMG to the gait cycle (beginning and ending at right heel strike) and averaged across gait cycles for each EMG electrode. We found (mean ± SD) 45.9 ± 18.9 baseline gait cycles per subject. The maximum value of the average time-warped gait cycle for each EMG electrode was used for normalization. Such peak gait cycle normalization has been shown to reduce intersubject variability compared with using maximum voluntary contractions (Yang and Winter, 1984). We epoched the normalized EMG activity around each perturbation onset, subtracted the baseline activity during the half-second before perturbation onset, and averaged across trials for each perturbation type. Based on these results, we averaged the perturbation-evoked EMG over a 0.3 s time window (0.2–0.5 after perturbation onset) for each subject to statistically compare peak EMG activity. The 2 × 4 repeated-measures ANOVAs analyzed intracondition main effects of muscle type (tibialis anterior, soleus, gastrocnemius, peroneus longus) and body side (left vs right). We also used a 2 × 2 repeated-measures ANOVA to test for intercondition main effects of physical task and perturbation type. *Post hoc* pairwise comparisons were performed using *t* tests, corrected for multiple comparisons using false discovery rate.

To test for the presence of adaptation effects during each trial, we calculated the pull force, peak EMG amplitude, and mediolateral marker position for the head and sacrum during the first and last minute of each trial. We performed 2 × 4 repeated-measures ANOVAs to look for significant main effects of trial type and adaptation (first minute vs last minute). *Post hoc* pairwise comparisons were performed using *t* tests with false discovery rate correction. All non-EEG statistics were calculated in R (R Core Team, 2017), with statistical significance determined if the *p* value was <0.05.

### EEG data processing

We processed all EEG data using custom EEGLAB scripts (Delorme and Makeig, 2004). EEG data were downsampled to 256 Hz, 1 Hz high-pass filtered, merged across all conditions, and referenced to the common median of all channels. We reduced 60 Hz line noise using Cleanline (Mullen, 2014). We rejected bad channels that had high SDs, had kurtosis >5 SDs, or were uncorrelated for >1% of the time (Kline et al., 2015; Luu et al., 2017b). We retained (mean ± SD) 111 ± 7 channels.

We further denoised the remaining EEG channels. To remove large mechanical artifacts, we used artifact subspace reconstruction (Mullen et al., 2013) with a threshold of 20 SDs, which has been used in a previous mobile EEG study (Artoni et al., 2017). We also performed selective low-pass filtering using ensemble empirical mode decomposition (Wu and Huang, 2009; Al-Subari et al., 2015) and canonical correlation analysis (Hotelling, 1936), similar to a technique used by Roy et al. (2017). This specifically targeted large high-frequency activity with low autocorrelation, such as muscle activity and line noise (Safieddine et al., 2012). We then rereferenced the data to the common average and interpolated the rejected channels to maintain a consistent montage across the head.

Next, we ran independent component analysis on the data using adaptive mixture independent component analysis (Palmer et al., 2006, 2008). Before this, we ran principal component analysis to ensure that the data sent into independent component analysis was full rank. We reduced down to 90 principal components for all subjects to maximize the ratio of data points to channels, which helps ensure that independent component analysis can properly separate sources of activity (Särelä and Vigário, 2003).

**Figure 2.**
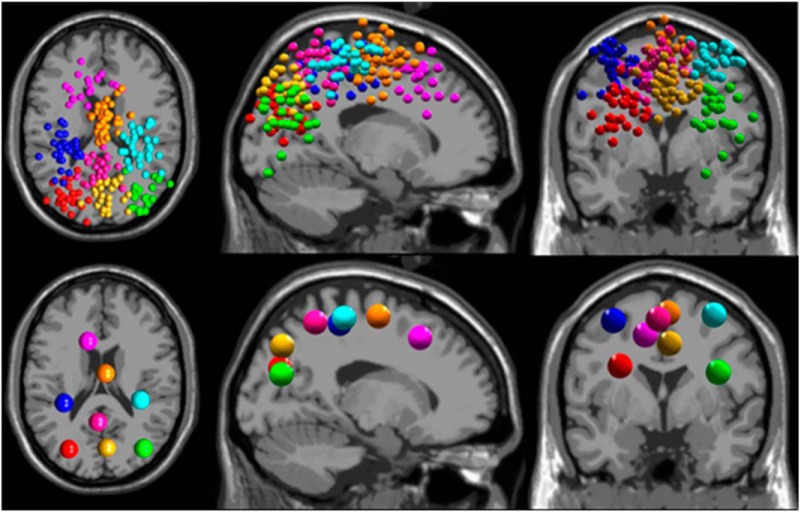
The resulting cortical dipoles corresponding to independent components are shown (top) for all subjects (*n* = 30), colored according to its corresponding cluster. Cluster centroids are shown (bottom) in axial (left), sagittal (middle), and coronal (right) views. Cluster coloring is as follows: left occipital, red; right occipital, green; posterior parietal, yellow; anterior parietal, pink; left sensorimotor, blue; right sensorimotor, cyan; supplementary motor, orange; and anterior cingulate, purple.

After independent component analysis, we fit each independent component to an equivalent dipole using DIPFIT2, retaining components that explained >85% of the scalp variance (Oostenveld and Oostendorp, 2002). We removed noncortical components based on visual inspection of dipole location and power spectra, retaining 240 total dipoles. These remaining cortical dipoles were clustered using k-means, with weights of 10 for dipole locations, 2 for power spectra, and 1 for scalp maps. Dipoles >3 SDs from the final clusters were placed in an outlier cluster. We analyzed clusters containing dipoles from more than half of the subjects (>15 subjects), which resulted in eight clusters (Fig. 2). These clusters were located in left occipital (18 subjects, 25 dipoles), right occipital (16 subjects, 22 dipoles), posterior parietal (23 subjects, 29 dipoles), anterior parietal (18 subjects, 23 dipoles), left sensorimotor (23 subjects, 25 dipoles), right sensorimotor (22 subjects, 30 dipoles), supplementary motor (27 subjects, 48 dipoles), and anterior cingulate (16 subjects, 18 dipoles) areas.

To analyze electrocortical activity during each trial, we calculated average EEG log power spectra. We compared spectral differences across trials by averaging spectral power into the following four frequency bands: theta (4–8 Hz), alpha (8–13 Hz), beta (13–30 Hz), and gamma (30–100 Hz). The 2 × 2 nonparametric permutation test repeated-measures ANOVAs were used to analyze the main effects of perturbation type and physical task for each frequency band, with 2000 permutations for each test. Further pairwise comparisons were performed using Wilcoxon rank-sum tests, corrected for multiple comparisons using false discovery rate. These statistics were performed in MATLAB 2013a (MathWorks), with significance at *p* < 0.05 for all tests.

We assessed perturbation-evoked electrocortical activity with EEG log time–frequency activity averaged across epochs, known as event-related spectral perturbations (ERSPs). We split the data into 2 s epochs (0.5 s before to 1.5 s after perturbation onset), resulting in (mean ± SD) 150 ± 1 epochs for stand pull, 145 ± 22 epochs for walk pull, 148 ± 7 epochs for stand rotate, and 149 ± 0 epochs for walk pull. We subtracted baseline activity for the half-second preceding perturbation onset. We used bootstrap statistics in MATLAB to determine significant differences from baseline, with a significant difference at *p* < 0.05. Nonsignificant values were set to 0. When calculating ERSPs, we took the median across trials instead of the mean to ensure that any large power fluctuations from a single trial did not skew the final ERSP results.

Because of the consistent spectral pattern across clusters, we were able to quantify the onset of each perturbation-evoked synchronization and desynchronization. We chose the largest contiguous region between 200 ms before and 500 ms after the perturbation onset, specifying frequency bands of 4–13 Hz for the synchronization and 8–30 Hz for the desynchronization. These bands were chosen based on the frequencies of the major ERSP fluctuations for both perturbation types. We defined the onset latency as the first time bin when the ERSP was outside of ±1 dB. This was performed on significance-masked ERSPs for every dipole within each cluster. For each cluster, we performed one-way Kruskal–Wallis tests to test for a significant effect of synchronization versus desynchronization onsets. We also performed two separate Kruskal–Wallis tests for significant effects of perturbation type (rotations vs pulls) and physical task (standing vs walking) for each of the synchronization and desynchronization onsets (five total tests per cluster). We performed these statistics in R, with significance at *p* < 0.05.

We also assessed neck muscle EMG recorded from two EEG external electrodes placed on the back of the neck, ∼5 cm above the seventh cervical vertebrae (C7) and 1 cm to either side. We referenced these externals to the EEG common-average reference and use artifact subspace reconstruction to remove any gross artifact. We epoched the muscle activity into the same 2 s epochs as the EEG data, calculating average EEG log power spectra and ERSPs using the same methods as for the EEG data. We performed the same ERSP significance masking and 2 × 2 nonparametric permutation test repeated-measures ANOVAs for the power spectra, with significance at *p* < 0.05. We have included statistical tables summarizing all statistical tests performed (Tables 1–3). Note that all pairwise comparisons used a false discovery rate correction (Benjamini and Yekutieli, 2001), and all *p* values presented for these comparisons are adjusted to keep the false-positive (α) threshold at 0.05.

**Table 1.** Statistical table for behavioral analyses

Measure	Data structure	Type of test	Power (parametric) or 95% confidence interval (nonparametric)
Pull force	Normal	2 × 2 Repeated-measures ANOVA	Pull direction, 0.568; physical task, 0.360; interaction, 0.059
Sacrum marker SD (Fig. 4A)	Normal	2 × 2 Repeated-measures ANOVA	Perturbation type, 1.00; physical task, 0.997; interaction, 0.358
Head marker SD (Fig. 4B)	Normal	2 × 2 Repeated-measures ANOVA	Perturbation type, 1.00; physical task, 0.998; interaction, 0.378
EMG intraconditions (Fig. 5)	Normal	2 × 4 Repeated-measures ANOVA	Stand Pull (Muscle type, 1.00; body side, 1.00; interaction, 0.730); Walk Pull (Muscle type, 1.00; body side, 0.054; interaction, 0.228); Stand Rotate (Muscle type, 0.206; body side, 0.088; interaction, 0.153); Walk Rotate (Muscle type, 0.217; body side, 0.233; interaction, 0.123)
EMG interconditions (Fig. 5)	Normal	2 × 2 Repeated-measures ANOVA	Perturbation type, 0.950; physical task, 1.00; interaction, 0.988
Behavioral adaptation (Fig. 6)	Normal	2 × 2 Repeated-measures ANOVA	Pull force (trial type, 0.698; adaptation, 0.589; interaction, 0.052); peak EMG (trial type, 1.00; adaptation, 0.379; interaction, 0.196); head marker SD (trial type, 1.00; adaptation, 0.111; interaction, 0.896); sacrum marker SD (trial type, 1.00; adaptation, 0.263; interaction, 0.174)

The data structure, type of statistical test used, and statistical power are shown for all behavioral statistical tests performed. We calculated two-way repeated-measures ANOVA power using the anova_stats() function from the sjstats library in R.

**Table 2.** Statistical table for ERSP onsets

Measure	Data Structure	Type of test	95% confidence interval
EEG ERSP onset latencies between synchronization/desynchronization (Fig. 10)	Non-normal	One-way Kruskal–Wallis test	Left occipital (sync, 52.7–105; desync, 182–242); right occipital (sync, 54.7–114; desync, 160–203); posterior parietal (sync, 23.4–70.3; desync, 176–234); anterior parietal (sync, 54.7–145; desync, 188–250); left sensorimotor (sync, 39.1–113; desync, 137–203); right sensorimotor (sync, 82.0–145; desync, 195–250); supplementary motor area (sync, 54.7–97.7; desync, 160–258); anterior cingulate (sync, 137–203; desync, 129–234)
EEG ERSP synchronization onset (Fig. 10)	Non-normal	Two one-way Kruskal–Wallis tests	Stand (left occipital, 39.1–105; right occipital, 54.7–112; posterior parietal, 7.81–54.7; anterior parietal, 7.81–97.7; left sensorimotor, 7.81–76.2; right sensorimotor, 31.2–113; supplementary motor area, 39.1–84; anterior cingulate, 113–211); walk (left occipital, 39.1–189; right occipital, 54.7–129; posterior parietal, 54.7–113; anterior parietal, 97.7–188; left sensorimotor, 82.0–176; right sensorimotor, 97.7–188; supplementary motor area, 82.0–121; anterior cingulate, 113–256); pull perturbation (Left occipital, 70.3–189; right occipital, 70.3–152; posterior parietal, 54.7–152; anterior parietal, 23.4–160; left sensorimotor, 15.6–89.8; right sensorimotor, 70.3–145; supplementary motor area, 7.81–39.1; anterior cingulate, 82.0–174); rotation perturbation (left occipital, 23.4–70.3; right occipital, 26.8–105; posterior parietal, 7.81–54.7; anterior parietal, 37.9–143; left sensorimotor, 39.1–160; right sensorimotor, 70.3–160; supplementary motor area, 113–176; anterior cingulate, 176–242)
EEG ERSP desynchronization onset (Fig. 10)	Non-normal	Two one-way Kruskal–Wallis tests	Stand (left occipital, 182–281; right occipital, 160–219; posterior parietal, 176–234; anterior parietal, 197–266; left sensorimotor, 145–219; right sensorimotor, 188–250; supplementary motor area, 176–309; anterior cingulate, 97.7–266); walk (left occipital, 137–219; right occipital, 145–227; posterior parietal, 176–234; anterior parietal, 129–281; left sensorimotor, 113–211; right sensorimotor, 160–273; supplementary motor area, 70.3–234; anterior cingulate, 54.7–266); pull perturbation (Left occipital, 82.0–273; right occipital, 84.0–219; posterior parietal, 105–234; anterior parietal, 189–316; left sensorimotor, 121–195; right sensorimotor, 160–250; supplementary motor area, 145–309; anterior cingulate, 99.8–281); rotation perturbation (left occipital, 188–242; right occipital, 188–219; posterior parietal, 196–234, anterior parietal, 176–250; left sensorimotor, 145–250; right sensorimotor, 188–258; supplementary motor area, 113–242; anterior cingulate, 54.7–242)

Data structure, type of statistical test used, and 95% confidence intervals are shown for ERSP onset. We calculated 95% confidence intervals using bootstrap statistics with 5000 replicates.

**Table 3.** Statistical table for EEG power analyses

Measure	Data structure	Type of test	95% confidence interval
EEG power spectra (Fig. 7)	Non-normal	2 × 2 Permutation repeated-measures ANOVA	
EEG ERSPs (Figs. 8, 9)	Non-normal	Bootstrap statistics	
Neck power spectra (Fig. 11)	Non-normal	2 × 2 Permutation repeated-measures ANOVA	
Neck muscle ERSPs (Fig. 11)	Non-normal	Bootstrap statistics	

The data structure and type of statistical test used are shown for EEG ERSP and power spectra statistical comparisons performed. We did not include power or confidence intervals due to the high number of comparisons performed..

## Results

### Marker SD and perturbation response

**Figure 3.**
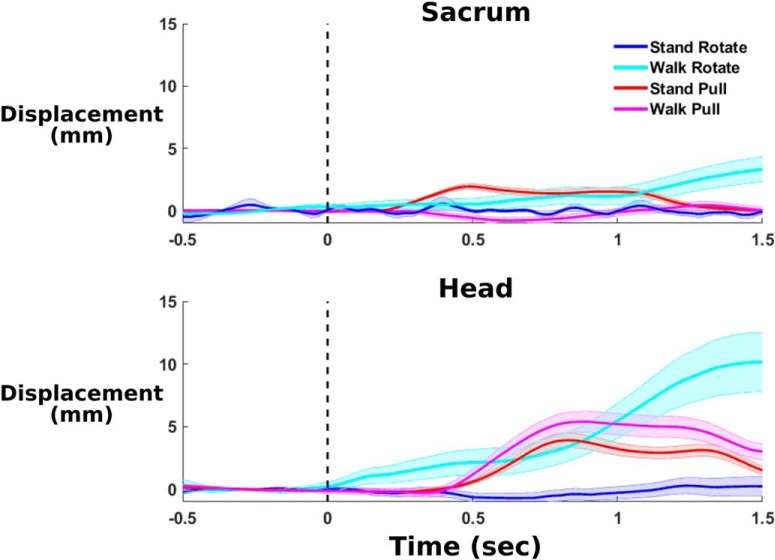
Average sacrum (top) and head (bottom) mediolateral displacement is shown for all conditions (*n* = 30), time locked to the perturbation onset at 0 s (shading shows SE). We rectified displacements to quantify average mediolateral movements away from the beam in either direction. We subtracted out baseline activity during the half-second before perturbation onset. Displacements of both markers stayed near to or <0.5 cm for the first second after the perturbation onset, indicating little consistent head or body mediolateral movement to the perturbation. This suggests limited motion artifact contamination in the EEG data.

Pull perturbations induced rapid mediolateral displacements in the subject’s head and torso, but visual perturbations led to a delayed head mediolateral displacement that was more prominent for walking compared with standing (Fig. 3). Mediolateral head and sacrum position changed starting at ∼400 ms after perturbation onset. In contrast, visual perturbations during standing induced no noticeable displacements in head and sacrum. Average head and sacrum displacements following perturbation onset were small, indicating that minimal motion artifact is present in the EEG (Fig. 3). Marker displacements were primarily <1 cm, suggesting little head and sacrum motion immediately following perturbation onset. The walk rotate trial appears to induce the largest deviation of the head, which is most pronounced ∼1 s after perturbation onset. This suggests a lack of consistent head motion immediately following perturbation onset. Based on this, we would expect limited motion artifact contamination in the EEG data.

**Figure 4.**
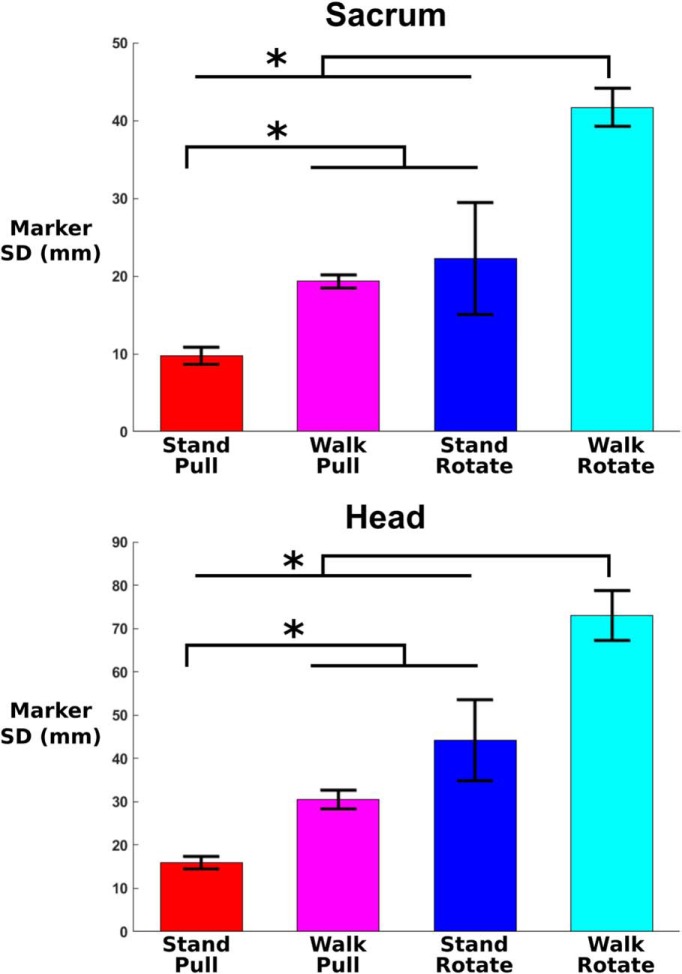
The average sacrum (top) and head (bottom) mediolateral SD for each entire trial is shown (*n* = 30; error bars show the SE). Asterisks indicate significant pairwise differences (*p* < 0.05). Both head and sacrum mediolateral sway indicate that subjects had the least side-to-side movement to pull perturbations during stance and the most movement to visual rotations during walking.

Across each entire trial, estimated sacrum and head mediolateral sway was notably increased during walk rotate and decreased during stand pull (Fig. 4). Our 2 × 2 repeated-measures ANOVA found significant main effects of perturbation type (sacrum, *p* = 1.62e-7; head, *p* = 1.09e-12) and physical task (sacrum, *p* = 8.08e-6; head, *p* = 3.47e-6) for both markers. The interaction terms were not significant. Pairwise comparisons for both markers found that walk rotate had a significantly increased SD compared with stand pull (sacrum, *p* = 2e-16; head: *p* = 2.1e-12), walk pull (sacrum, *p* = 2.7e-12; head, *p* = 1.1e-9), and stand rotate (sacrum, *p* = 0.004; head, *p* = 0.003). The marker SD was also significantly decreased during stand pull compared with walk pull (sacrum, *p* = 4.5e-11; head, *p* = 1.2e-8) and stand rotate (sacrum, *p* = 0.047; head, *p* = 1.7e-3). We found no other significant differences. Note that this estimated sway is the average across the entire trial and does not reflect the perturbation-evoked displacement responses shown in Figure 3.

### Pull force results

We found no differences in pull perturbation force between walking and standing trials, and between right and left pulls. Pull forces to the subject’s left side were (mean ± SD) 16.73 ± 6.56 N during standing and 15.76 ± 6.23 N during walking. Pull forces to the subject’s right side were 15.38 ± 2.15 N during standing and 14.00 ± 2.54 N during walking. While we found a significant main effect of pull direction (*p* = 0.035), we found no significant pairwise differences in pull direction during standing (*p* = 0.420) and walking (*p* = 0.420). We found no significant main effect of physical task (*p* = 0.112), and the interaction term was also not significant (*p* = 0.778).

### EMG perturbation response

**Figure 5.**
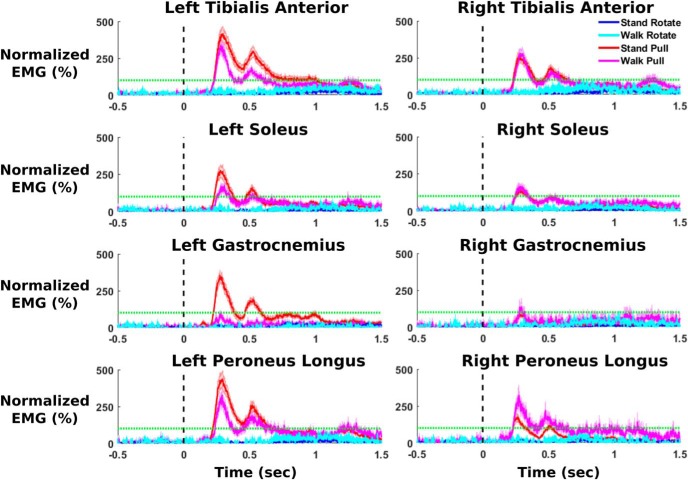
The average rectified EMG activity is shown for eight lower leg muscles across all trials (*n* = 30), time locked to the perturbation onset at 0 s (shading shows the SE). The activity of each muscle was normalized to peak EMG activity during the 15 s of walking before perturbation onset during the walk pull condition. The horizontal green line indicates this 100% peak EMG activity during walking. We subtracted the off-baseline activity during the half-second before perturbation onset. Pull perturbations show clear increases in muscle activity following perturbation onset, with substantially increased left leg muscle activity to pull perturbations administered during standing. This is especially noticeable between the left and right medial gastrocnemius.

We found substantial differences in peak EMG activity across muscles following perturbation onset, along with notably increased left leg EMG compared with right leg EMG during stand pull. The average EMG perturbation response is shown in Figure 5. The 2 × 4 repeated-measures ANOVA for stand pull found significant main effects for muscle type (*p* = 3.68e-9), body side (*p* = 2e-16), and their interaction (*p* = 0.027). Pairwise comparisons showed significant increases in all left-side muscles compared with right-side muscles (tibialis anterior, *p* = 0.0087; gastrocnemius, *p* = 5.17e-11; peroneus longus, *p* = 5.58e-7), except for soleus (*p* = 0.065). Across muscles, we found significantly decreased soleus activity compared with tibialis anterior (*p* = 6.94e-6) and peroneus longus (0.021) activity. Gastrocnemius peak EMG was also significantly decreased compared with tibialis anterior (*p* = 1.32e-6) and peroneus longus (*p* = 0.002). Tibialis anterior peak EMG was also significantly greater than peak peroneus longus EMG (*p* = 0.039). No other significant pairwise comparisons were found. For walk pull, we found a significant main effect of muscle type (*p* = 4.38e-14), with significantly decreased soleus and gastrocnemius EMG compared with tibialis anterior (soleus, *p* = 7.12e-6; gastrocnemius, *p* = 3.17e-13) and peroneus longus (soleus, *p* = 3.66e-5; gastrocnemius, *p* = 8.26e-13). Peak gastrocnemius EMG was significantly decreased compared with soleus (*p* = 1.65e-8). No other comparisons were significant. ANOVAs for stand rotate and walk rotate found no significant effects.

For EMG across all four conditions, pull perturbations substantially increased peak EMG compared with visual perturbations, with notable differences in muscle activity between standing and walking. Our 2 × 2 repeated-measures ANOVA across all four conditions showed a significant main effect of physical task (*p* = 3.32e-4), a significant main effect of perturbation type (*p* = 2e-16), and a significant interaction term (*p* = 2.53e-5). For pairwise comparisons, we found significantly increased peak EMG activity between the pull and rotate conditions for almost all muscles. The only exception was that the right gastrocnemius muscle did not significantly differ between walk pull and both stand rotate (*p* = 0.077) and walk rotate (*p* = 0.171). We found significantly increased EMG during stand pull compared with walk pull for left tibialis anterior (*p* = 0.008), left soleus (*p* = 0.025), left gastrocnemius (*p* = 2.17e-11), and left peroneus longus (*p* = 0.003). In contrast, we found significantly increased right peroneus longus (*p* = 0.002) EMG during walk pull compared with stand pull.

### Behavioral adaptation during each trial

**Figure 6.**
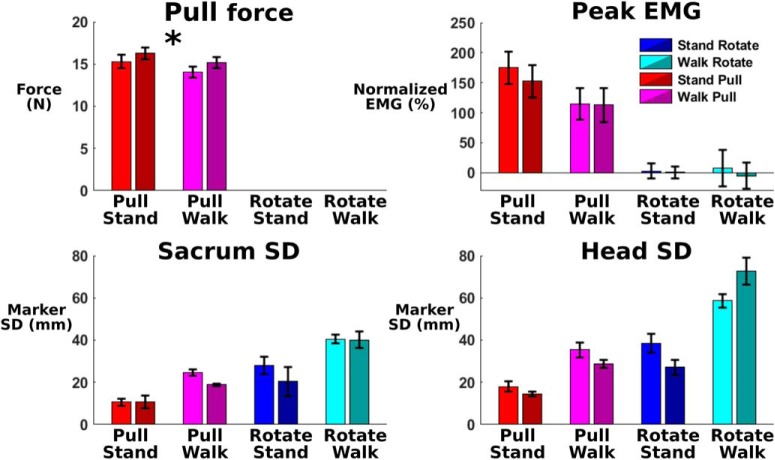
The average and SE of the first (lighter color) and last (darker color) minute of each 10 min trial are shown for behavioral measures (*n* = 30). Because the pull force could be calculated only during the pull perturbation, there are no values during the rotation perturbations. We only found a significant difference between the first and last minute for pull force (denoted by asterisk; repeated-measures ANOVA, *p* = 0.031), although no pairwise comparisons were significant. We found no other significant adaptation effects for the other measures. Our results indicate that minimal adaptation effects were present.

There was no notable adaptation in any behavioral measure from the first to the last minute of each trial. Comparing the first minute to the last minute of each 10 min trial showed no statistical differences in SD of sacrum (*p* = 0.187) and head (*p* = 0.474) marker mediolateral position or pooled EMG amplitude across muscles (*p* = 0.099), as shown in Figure 6. We did find a significant adaptation of pull force (*p* = 0.031), but no pairwise comparisons were significant (*p* = 0.200 for walk pull early vs stand pull late; *p* = 0.410 for all other comparisons). There was also a significant interaction between trial and adaptation effects for the head mediolateral SD (*p* = 0.004). *Post hoc* pairwise *t* tests found no significant adaptation effects for head mediolateral SD during stand pull (*p* = 0.208), walk pull (*p* = 0.141), stand rotate (*p* = 0.118), and walk rotate (*p* = 0.118). Adaptation effects appeared minimal during each trial, indicating that each trial can be considered reasonably consistent from beginning to end.

### EEG power spectra

**Figure 7.**
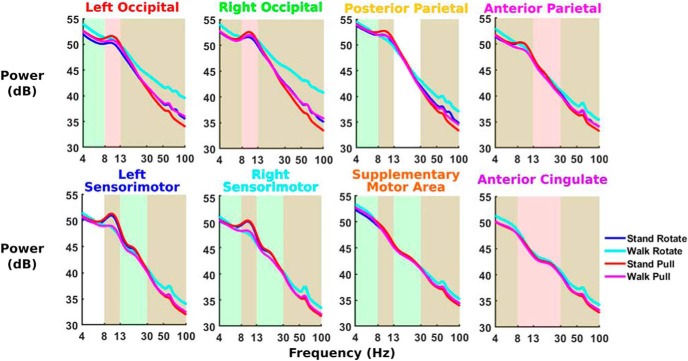
The average EEG power spectra are shown for each trial (*n* = 30), with log scaling along the *x*-axis. Shading reflects significant 2 × 2 ANOVA main effects. Green shading indicates a significant main effect of physical task (standing vs walking), red shading indicates a significant main effect of perturbation type (rotation vs pull), and brown shading indicates that both main effects are significant. We found significant increases in theta and gamma power during walk rotate compared with the other three conditions, primarily in occipito-parietal areas. We also found significant increases in alpha and beta power during standing compared with walking in sensorimotor areas.

We found significantly increased theta spectral power during walk rotate compared with all other conditions across multiple areas (Fig. 7). Walk rotate showed significantly increased theta power in right occipital, left occipital, anterior parietal, and anterior cingulate compared with all other conditions (*p* = 5.0e-4 for all).

Alpha and beta power were substantially increased during standing conditions compared with walking in several cortical areas. Left and right sensorimotor areas showed significantly increased alpha and beta spectral power during stand pull and stand rotate compared with walk pull and walk rotate, respectively (*p* = 5.0e-4 for all). We also found significantly increased alpha power during both standing conditions compared with their corresponding walking conditions in posterior parietal (*p* = 5.0e-4 for both). In addition, we found significantly increased alpha and beta power in supplementary motor area during stand pull compared with walk pull (*p* = 5.0e-4), but no significant difference between conditions with the visual rotation.

In gamma band, we primarily found increased spectral power during walk rotate and decreased spectral power during stand pull. Across all clusters, walk rotate had significantly increased gamma power compared with all other conditions (*p* = 5.0e-4 for all). We also found significantly decreased gamma power for stand pull compared with all other conditions in left occipital, right occipital, and posterior parietal (*p* = 5.0e-4 for all).

### EEG ERSPs

**Figure 8.**
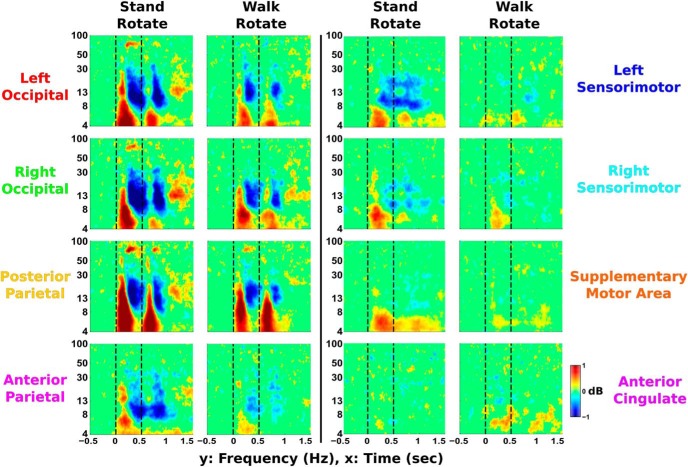
EEG ERSPs are shown for the visual rotation perturbations during standing and walking (*n* = 30). Significant increases in spectral power relative to baseline (the 500 ms before perturbation onset) are shown in red and are referred to as synchronization. Significant decreases in power relative to baseline are displayed in blue and are referred to as desynchronization. Vertical lines indicate perturbation onset and termination at 0 and 0.5 s, respectively. Nonsignificant differences from baseline (bootstrap statistics, *p* ≥ 0.05) were set to 0 dB (green). Occipito-parietal areas showed the largest spectral fluctuations, while the anterior cingulate had few changes in spectral power.

ERSP plots for visual perturbations show theta synchronization immediately after perturbation onset followed by alpha–beta desynchronization (Fig. 8). A similar pattern of time–frequency activity occurs immediately following perturbation termination. This pattern is strongest in the left occipital, right occipital, and posterior parietal areas, with weaker patterns of synchronization and desynchronization seen in other cortical clusters.

**Figure 9.**
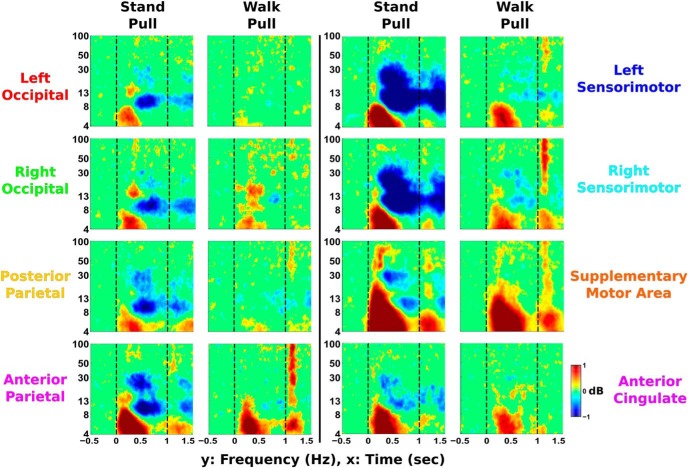
EEG ERSPs are shown for the pull perturbations during standing and walking (*n* = 30). Significantly increased spectral power compared with baseline (the 500 ms before perturbation onset) is displayed in red and is known as synchronization. Significantly decreased power compared with baseline is shown in blue and is referred to as desynchronization. Vertical lines indicate perturbation onset and termination at 0 and 1 s, respectively. Nonsignificant differences from baseline (bootstrap statistics, *p* ≥ 0.05) have been set to 0 dB (green). Centro-frontal motor areas show large fluctuations in spectral power following perturbation onset, with the greatest theta synchronization in supplementary motor area. Alpha–beta desynchronization (8–30 Hz) is most prominent in left and right sensorimotor clusters.

Pull perturbation ERSPs show a similar pattern of theta synchronization followed by alpha–beta desynchronization during perturbation onset and termination (Fig. 9), but primarily located in different cortical areas compared with the visual perturbation. Theta synchronization appears in sensorimotor and anterior cingulate areas, with the strongest activity in the supplementary motor area. Large alpha–beta desynchronization also occurs in these areas, with the strongest activity in left and right sensorimotor areas. Similar time–frequency patterns with weaker strength were seen in occipito-parietal areas.

**Figure 10.**
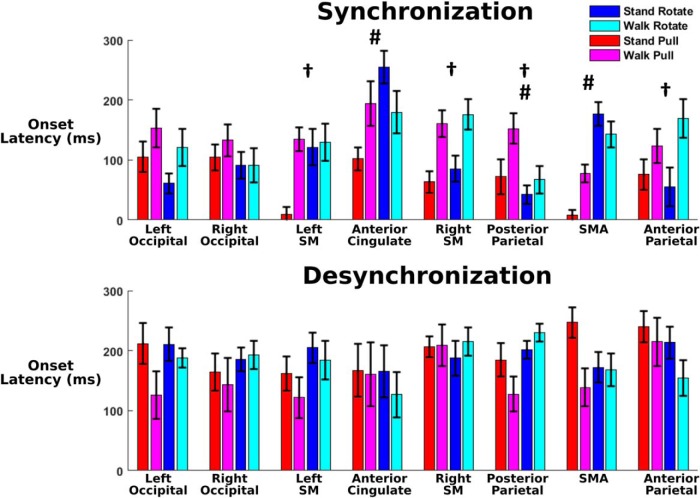
The onset latencies of ERSPs across each cluster are shown for the theta–alpha synchronization (top) and alpha–beta desynchronization (bottom), with error bars showing the SE. We have indicated significant one-way Kruskal–Wallis main effects of perturbation type (#rotation vs pull) and physical task (†standing vs walking). Left and right SM indicate left and right sensorimotor areas, and SMA indicates supplementary motor area. Most significant effects were found in centro-frontal motor areas during synchronization onset. We found significantly increased desynchronization onset latency compared with synchronization onset latency in all clusters except anterior cingulate.

ERSP synchronization onset occurred notably before desynchronization in most cortical clusters, with differences in synchronization onset across trials in multiple sensorimotor areas (Fig. 10). In all clusters except the anterior cingulate (*p* = 0.615), we found significantly earlier synchronization onset compared with desynchronization onset (left occipital, *p* = 5.85e-5; right occipital, *p* = 6.04e-5; posterior parietal, *p* = 7.56e-10; anterior parietal, *p* = 1.14e-5; left sensorimotor, *p* = 2.70e-4; right sensorimotor, *p* = 1.30e-6; supplementary motor area, *p* = 1.24e-5). In addition, we found a significant effect of perturbation type during synchronization onset in posterior parietal (*p* = 0.019), supplementary motor area (*p* = 4.18e-11), and anterior cingulate (*p* = 0.024). We also found a significant effect of physical task during synchronization onset in posterior parietal (*p* = 0.006), anterior parietal (*p* = 0.008), left sensorimotor (*p* = 0.005), and right sensorimotor (*p* = 1.88e-4) areas. For desynchronization onset, we found no significant main effects of perturbation type or physical task.

### Neck muscle EMG

**Figure 11.**
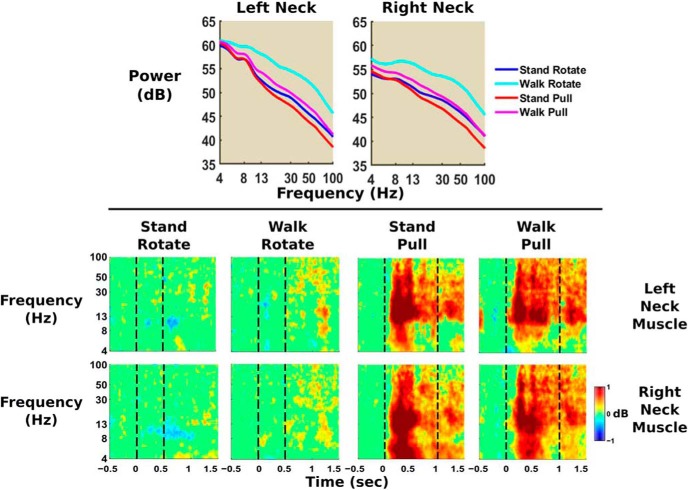
Average power spectra (top) and median ERSP plots (bottom) are shown for left and right neck muscle EEG electrode locations (*n* = 30). Power spectra shading indicates that there were significant 2 × 2 repeated-measures ANOVA effects of perturbation type and physical task across all frequency bands. The neck muscle power spectra for the walk rotate condition was noticeably higher than the other three conditions for both neck electrodes. Significantly increased spectral power compared with baseline (the 500 ms before perturbation onset) is displayed in red and significantly decreased power compared with baseline is shown in blue. Vertical lines indicate perturbation onset at 0 s and perturbation termination at 0.5 or 1 s, depending on the perturbation type. We set nonsignificant differences from baseline (bootstrap statistics, *p* ≥ 0.05) to 0 dB (green). Based on the ERSPs, only the pull perturbations appear to immediately increase neck muscle activity. Neck muscle activity only showed up as increased spectral power on the ERSP, and not as decreased spectral power.

Neck muscles showed substantially increased spectral power in the walk rotate condition and perturbation-evoked power increased only during the pull perturbations (Fig. 11). We found significantly increased beta and gamma power during walk rotate compared with the other conditions and significantly decreased beta and gamma power for stand pull compared with all other conditions (*p* = 5.0e-4 for all). ERSP plots show significantly increased neck muscle power immediately following pull perturbation onsets, primarily in beta and gamma frequency bands. In contrast, we found few power fluctuations during the visual rotation perturbations.

## Discussion

We were able to identify robust electrocortical fluctuations in response to perturbations that challenged balance. We found transient theta synchronization and alpha–beta desynchronization following perturbation onset, as hypothesized. This spectral activity increased in occipito-parietal areas following visual perturbations, whereas physical pulls increased activity in sensorimotor areas, as hypothesized. Surprisingly, we found little activity in the anterior cingulate following visual perturbations. This study demonstrates that analysis of spectral power fluctuations in clusters of electrocortical sources can provide considerable insight into the networks and functional activity related to sensorimotor tasks (Makeig, 2002; Gramann et al., 2011).

### Body sway and muscle response

Body and head sway notably increased during walking and during virtual reality use, indicating reduced stability (Fig. 4). We expected to find increased body and head sway during walking compared with standing because walking involves dynamic balance. In addition, body and head motion increased when subjects wore the virtual reality headset for the visual perturbations compared with unaltered viewing during the pull perturbations. Based on previous studies, this may indicate decreased stability when wearing a head-mounted display (Kelly et al., 2008; Robert et al., 2016). In our virtual reality setup, reduced stability could have been caused by a reduced field of view, low latency, or the location of the webcam below eye level.

Our lower leg EMG results showed that physical pull perturbations induced a robust muscle response while visual perturbations did not elicit a consistent muscle response (Fig. 5). This is not surprising because the pull perturbations physically attempt to move the subject mediolaterally, necessitating a muscular response. The visual perturbation does not physically alter each subject’s movement, instead relying on the disruption of visual input to require a balance response. Physical mediolateral perturbations induce a reflex response, where the ankle muscles attempt to brake side-to-side motion by cocontracting (Hof and Duysens, 2018). This quick muscle response to physical pull perturbations highlights the importance of sufficient muscle strength to maintain stability (Papa et al., 2015).

During the pull perturbations, muscle activity was greatest in the peroneus longus and tibialis anterior, with a notable asymmetry between left and right leg muscles during standing. The tibialis anterior, peroneus longus, and medial gastrocnemius have been shown to be important in mediolateral body stabilization, while soleus activity may be more active during posterior perturbations (Henry et al., 1998). Interestingly, muscle activity during stand pull was notably asymmetrical, with left leg muscle activity higher than the corresponding right leg muscles, especially for the medial gastrocnemius. Subjects stood with their left leg in back, suggesting that the back leg was more involved in stabilization. The large increase in the left gastrocnemius muscle may indicate the recruitment of larger leg muscles to help stabilize the body during balance. It would be interesting to see whether this asymmetrical muscle response also occurs between the front and back foot during walking, but this would require timing the perturbation to occur during the double support phase.

### EEG power spectra

Electrocortical spectral power showed increased alpha power during standing compared with walking, likely reflecting differences in motor readiness (Fig. 7). We found this increased alpha power during standing compared with walking in left/right occipital, left/right sensorimotor, anterior parietal, posterior parietal, and supplementary motor area, with the largest differences in sensorimotor areas. Alpha power has been shown to decrease when walking compared with standing (Presacco et al., 2011; Youssofzadeh et al., 2016). Alpha power can also decrease when performing cognitively engaging tasks such as walking in an interactive virtual environment (Wagner et al., 2014) and closed-loop brain–computer interface control of a virtual avatar while walking (Luu et al., 2017a).

In addition, we found significantly increased theta power across multiple clusters when subjects were exposed to visual perturbations while walking, possibly indicating a cognitive response to challenging balance conditions. Body and head sway substantially increased during walk rotate compared with all other conditions, suggesting that this condition challenged balance the most. Increased theta power has been seen during tasks requiring balance (Slobounov et al., 2009; Sipp et al., 2013) and can correspond to more challenging balance tasks (Hülsdünker et al., 2015; Youssofzadeh et al., 2016). Increased theta power during walk rotate seems to provide a cognitive indicator that balance difficulty increased.

In the gamma band, we found increased power during walking visual rotations and decreased power during standing physical pull perturbations for most brain areas. Gamma power differences appeared most pronounced in left occipital, right occipital, and posterior parietal. These power spectra differences align quite well to the head and body sway estimates (Fig. 4), with stand pull having the lowest sway and walk rotate having the highest. Gamma power has been implicated in active cortical processing (Başar et al., 2001) and can increase with greater instability (Slobounov et al., 2009). It is possible that neck muscle activity contaminated the occipital and posterior parietal clusters as they are closest to the back of the head where the neck muscles are located. However, there was virtually no neck muscle activation in response to the visual rotation and very strong neck muscle activation in response to the physical pull perturbation (Fig. 11). In contrast, the visual rotation had the greatest gamma power in occipital and posterior parietal clusters, and physical pull perturbations had low gamma power in occipital and posterior parietal clusters. These observations strongly suggest that our signal processing adequately removed neck muscle activity from the brain sources. Even for the physical pull perturbations when there was clear neck muscle activity, the neck ERSP showed frequencies primarily >13 Hz and remained fairly consistent after perturbation onset. The brain source synchronizations of interest were all <13 Hz and occurred within the first half-second after perturbation onset. For these reasons, it does not seem likely that the neck muscle electrical activity affected our results.

### Perturbation-evoked EEG

During the pull perturbations, large theta synchronization was seen in sensorimotor and supplementary motor areas (Fig. 9). This initial synchronization has been shown to be similar to the N1 peak seen during averaged event-related EEG activity following balance perturbations (Varghese et al., 2014). This N1 activity tends to be widespread, with strongest activity localized to the supplementary motor area (Marlin et al., 2014), which also shows the greatest theta synchronization in our study. N1 activity has been shown to be present despite changes in task (Quant et al., 2004). Similarly, theta synchronization in our data appears to show up in most clusters for both perturbation types and is well conserved between standing and walking. In addition, theta synchronization onset latency was notably altered in centro-frontal motor areas based on the type of perturbation and whether subjects stood or walked. Previous research (Ahmed, 2005) has shown that the brain uses an internal model and identifies loss of balance if body motion diverges too many SDs from the previous behavior. Walking involves more baseline movement than standing, which could notably increase this SD threshold used by the brain and potentially result in the delayed theta synchronization seen during walking. This increased threshold may be due to the increased mediolateral sway seen during walking. Interestingly, the single N1 EEG peak in young adults has been found to be delayed and more prolonged in older adults, especially in older adults with reduced mobility (Duckrow et al., 1999). This suggests that theta band synchronization may be useful for studying cognitive deterioration in balance performance.

Although the pull perturbations during standing elicited asymmetrical electrocortical onset times and leg muscle response amplitudes, these are likely unrelated to each other. We found a substantial increase in left leg muscle response compared with the right leg during pull perturbations while standing, likely due to the greater use of the back leg for maintaining balance. Similarly, the EEG synchronization onset during this trial (Fig. 10) showed a notable decrease in onset latency in left sensorimotor area compared with right sensorimotor area. Previous research during loss of balance showed that the left sensorimotor area was more active than the right sensorimotor area, regardless of the direction in which balance was lost and although leg muscle activity differed based on direction (Sipp et al., 2013). The authors concluded that the left sensorimotor area was the earliest electrocortical indicator in which balance is lost, which agrees with our findings. In another study (Bruijn et al., 2015), beta power in left premotor area was modulated during stabilized versus unaltered walking, while remaining unaffected in the right premotor area. Diffusion tensor imaging of older adults found significant correlations between stability measures and left hemisphere corticospinal tracts, with no significant correlations for right corticospinal tracts, suggesting corticospinal lateralization when maintaining stability (Bruijn et al., 2014). This may be explained by left hemisphere dominance in right-handed humans during a variety of skilled movements (Serrien et al., 2006), potentially implicating subjects’ handedness in inducing asymmetrical cortical results. These studies suggest that our subjects’ asymmetrical electrocortical onset times are likely influenced by brain laterality during balance control, not stance position, but further research is needed to verify this.

We also found large alpha–beta desynchronization in sensorimotor areas following the pull perturbation while standing, likely reflecting changes in motor readiness and decreased motor inhibition. This is further evidenced by the largest alpha–beta desynchronization during standing occurring in sensorimotor areas, which also showed the large increases in alpha–beta spectral power during standing compared with walking. Similar transient alpha–beta desynchronization has been shown previously (Seeber et al., 2014; Luu et al., 2017a). It has also been suggested that beta desynchronization may reflect the brain detecting a change from the status quo (Engel and Fries, 2010). During the visual rotations, beta desynchronization may indicate a change in the status quo due to conflict between visual and vestibular inputs and conflict between visual and proprioceptive inputs. Such alpha–beta frequency fluctuations may not readily correspond to averaged event-related activity, indicating that time–frequency decomposition can provide useful additional information.

The similarity in time–frequency patterns between visual and physical perturbations suggest a common electrocortical signature due to sensorimotor conflict (Figs. 8, 9). We were able to determine that low-frequency synchronization consistently occurred before higher-frequency desynchronization in most cortical areas, suggesting a similar pattern to sensorimotor perturbations. This pattern in our data is similar to that seen during visual conflict tasks using EEG (Jiang et al., 2018) and local field potential recordings in the subthalamic nucleus (Zavala et al., 2016; Hell et al., 2018). All three of these studies recorded similar theta and beta oscillations in the cortex, indicating an important connection between the subthalamic nucleus and cortex during conflict. This seems to warrant further exploration, especially due to the importance of the subthalamic nucleus in Parkinson’s disease (Collomb-Clerc and Welter, 2015). Based on the similar patterns elicited by visual and pull perturbations, one might expect notable differences in electrocortical activity using visual conflicts on patients with Parkinson’s disease compared with healthy adults, especially if they have freezing of gait symptoms (Gilat et al., 2013; Matar et al., 2013). Despite our study being limited to healthy, young adults, there seems to be enough evidence to suggest that similar time–frequency, perturbation-evoked EEG activity should be studied in patient populations. While Parkinson’s disease has been primarily associated with basal ganglia dysfunction (Blandini et al., 2000), dual-task studies have indicated that cortical activity is also affected (Yogev et al., 2005; Salazar et al., 2017), making EEG a potentially relevant recording site. It is also interesting to note similarities between our perturbation time–frequency pattern and the gait-related time–frequency pattern seen in other studies during foot–ground contact and initial stance (Gwin et al., 2011; Seeber et al., 2014). Gait time–frequency patterns usually show theta synchronization during heel strikes, which is similar to the synchronization we found after perturbation onset. This theta synchronization during foot–ground contact may indicate increased sensorimotor processing due to increased instability during stepping.

We were surprised by the lack of spectral fluctuations in anterior cingulate following the visual rotation (Fig. 8). We had hypothesized that there would be large occipito-parietal spectral fluctuations for the visual rotation condition, but we still expected some spectral fluctuations in the anterior cingulate based on past balance studies (Slobounov et al., 2009; Sipp et al., 2013) and gait (Gwin et al., 2011; Luu et al., 2017a). We did see anterior cingulate activity during pull perturbations, which was likely a more similar match to the previous studies. One interpretation for the lack of anterior cingulate spectral fluctuations after the visual rotations is that the anterior cingulate is primarily focused on maintaining balance and changes to physical posture. The posterior parietal and occipital areas may be primarily responsible for resolving visual conflict, so no further processing by the anterior cingulate is needed. The anterior cingulate has been shown to be active during error monitoring of visual conflicts (Gehring and Knight, 2000; van Veen and Carter, 2002), but it may depend on how the anterior cingulate defines errors (Carter, 1998). It is also worth noting that most visual flanker or Stroop tasks to analyze anterior cingulate activity require a motor response, whereas our visual perturbation did not necessitate a physical response. If the visual perturbations led to a step-off from the beam, we would expect to see anterior cingulate activity. Further research is needed, but this highlights the importance of measuring electrocortical activity during more real-world movements.

While we did not find consistent cortical sources in the prefrontal area, this should not necessarily be interpreted as the prefrontal areas being uninvolved with the perturbation response. On the contrary, multiple fNIRS studies have found increased prefrontal oxygenation during challenging balance tasks (Basso Moro et al., 2014; Ferrari et al., 2014) and dual tasking (Mirelman et al., 2014; Mahoney et al., 2016). It is possible that artifacts from eye movements and blinks in the EEG may have made it more challenging for independent component analysis to separate out prefrontal sources.

### Perturbation magnitude

A limitation of the study is the use of only one perturbation magnitude for the visual rotation and physical pull perturbations. In an ideal world, we could have conducted a range of different magnitudes of visual rotation (e.g., 5°, 10°, 20°, 40°, and 90° of rotation) and physical pull forces (e.g., 5, 10, 15, 25, and 50 N). This would have provided information about the relationship between perturbation size and the electrocortical dynamics timing and amplitude. Previous studies examining scalp EEG during perturbations to standing have found that either increasing the perturbation magnitude or shortening the perturbation duration can increase the low-frequency electrocortical response, with no differences in electrocortical timing (Dietz et al., 1985; Staines et al., 2001; Mochizuki et al., 2010). While these studies did show some scaling of electrocortical responses with perturbation magnitude, the relationship was less than proportional. Given that there was no effect on electrocortical timing in the previous studies, it suggests that measuring only one perturbation magnitude per condition was not a major weakness to our results. It may also be worth conducting future studies at faster walking speeds than 0.22 m/s. We did not include faster gait speeds because we did not want to add an extra confound into the experiment. Faster speeds increase the intersubject variability in balance performance as the task is more difficult. Choosing 0.22 m/s also makes it easier to compare our results with those of previous studies that have used the same speed (Domingo and Ferris, 2010, 2009; Sipp et al., 2013). However, previous studies have found no difference in cortical and muscular responses to cognitive dual tasking at various gait speeds, so the effect of gait speed may be minimal (Kline et al., 2014; Meester et al., 2014). Future studies that examine the patient population will likely want to examine scaling of the perturbation magnitude as they can have reductions in sensorimotor function that make detection of the perturbation different from neurologically intact subjects.

### EEG motion artifact

While any EEG study during human movement includes concerns about artifact contamination, motion artifacts appeared to have minimal effects on our results. A motion artifact has been shown to have differential effects across the head during walking (Kline et al., 2015). In contrast, our EEG ERSPs and power spectra were quite consistent between left and right clusters in occipital and sensorimotor areas (Figs. 8, 9). Such symmetry provides strong evidence against a motion artifact being present. In addition, we do not see broadband activity during perturbation onset, which is a hallmark of the motion artifact during EEG gait experiments (Oliveira et al., 2017a). We also validated our EEG hardware and signal-processing techniques against motion artifacts using an electrical head phantom and motion platform, as was done in previously published work (Oliveira et al., 2016). We are very confident that the EEG is not affected by motion artifacts, given this validation. Furthermore, the visual rotations do not elicit consistent head movement, especially during standing (Fig. 3). While some motion from the pull perturbations was expected, the head movement immediately following perturbation onset was at most 0.5 cm on average. Little consistent head motion appeared to be present, and the effects of inconsistent motion artifacts were likely reduced by averaging. In addition, taking the median across trials for the ERSPs instead of the mean likely prevented any inconsistent artifacts skewing the resulting ERSP. Using the median may be useful in future EEG studies, especially if motion artifacts are a concern.

### Conclusions

By testing subjects with brief visual rotation and physical pull perturbations, we were able to identify a highly conserved electrocortical time–frequency pattern, but in different brain regions. This pattern was strongest in occipito-parietal areas during visual perturbations and strongest in sensorimotor areas during pull perturbations. Such a common time–frequency signature may be important in assessing balance dysfunction and improving our understanding of balance control in individuals with mobility disorders.

## Synthesis

Reviewing Editor: Trevor Drew, University of Montreal Decisions are customarily a result of the Reviewing Editor and the peer reviewers coming together and discussing their recommendations until a consensus is reached. When revisions are invited, a fact-based synthesis statement explaining their decision and outlining what is needed to prepare a revision will be listed below. The following reviewer(s) agreed to reveal their identity: Daniel Marigold.

Reviewer 1

This manuscript provides novel insights into human EEG-based cortical responses to visual and physical balance perturbations during tandem standing and treadmill beam-walking. Visual perturbations consisted of brief field-of-view rotations using virtual reality goggles, whereas physical perturbations consisted of medial-lateral waist pulls. The authors show time-frequency fluctuations in different frequency bands (e.g., theta, beta, gamma) depending on the cortical area and perturbation type. The results are interesting and add to a growing body of literature using high-density EEG to study brain function during motor tasks. However, I have several comments the need to be addressed.

Major comments:

1. In the introduction, the rationale for using visual perturbations could be stronger. Although I can appreciate that this allows the authors to better understand how the brain uses vision, there is no mention of the sensory conflict this manipulation creates. Specifically, the rotations would create a temporary conflict between vision and vestibular input as well as between vision and proprioceptive input. The authors could also return to this idea in the discussion section in relation to their results.

2. Providing more specific hypotheses (with a rationale for them) would strengthen the introduction. The hypothesis (lines 83-85) is rather vague.

3. To make inter-muscle and inter-subject muscle comparisons, as done in the present study, it is standard to normalize EMG activity (for example, to peak activity during a control condition or to an MVC). How was EMG normalized in this study? Or was it? If not, what is the rationale for this and how may have this influenced your results?

Minor comments:

Line 29, significance statement: since the authors did not actually test this source, it is misleading to make this claim and thus, it should be removed from this section.

A gait speed of 0.22 m/s is extremely slow. Although I understand that this is because subjects are walking on a balance beam, how would faster gait speeds affect the results?

What was the rationale for having subjects walk with crossed arms? This is very unnatural.

Lines 138-144: what about neck EMG?

Line 181: what do the authors mean when they say “false discovery rate correction for multiple comparisons”? Please provide more details and/or a reference.

Lines 274-300: There is minimal to no head and sacrum marker displacement for the stand rotate condition (shown in Figure 3), yet this condition shows the second largest SD (displayed in Figure 4). Please clarify, as perhaps I am missing something.

Line 348: to which muscle(s) for EMG amplitude are you referring?

Line 492 and 496: These two sentences appear to say the same thing. Please revise this paragraph.

Lines 521-523: What does this (i.e., “increased cortical threshold detecting perturbations to balance”) mean?

Line 542: insert ‘to’ between ‘similar’ and ‘that’.

Figures 8 and 9: The text for the brain areas in the latter is in color (and correspond to the clusters in an earlier figure). However, the text in the former is in black. Should this not also be in color?

Figure 10: Line 918 in caption: remove ‘indicates’. Also, state whether the error bars are reported as SE or SD.

Confidential Review Collaborator

Reviewer 2

The authors analyzed and compared electrocortical (EEG)l activity during visual and physical perturbations to standing balance and walking. The authors found differential changes in theta ERS and alpha/beta ERD in occipito-parietal areas during visual perturbations and in sensorimotor and anterior cingulate areas during pull perturbations. Overall, this is a well designed study; however there are some MAJOR weaknesses that need to be addressed as well as some minor criticisms that the authors should consider as well.

Significance:

L26-27: Please rewrite this sentence for improved clarity (the phrase “physical perturbation” is repeated in the sentence)

Introduction:

L65-66: Please provide some examples for “physical manipulations” and “sensory perturbations”, suggesting how they are different?

L70-71: “increase in EEG spectral power” in what bands?

Also, clarify what population(s) are discussed in the literature review in the Introduction.

L79-80: Mention the study population of your interest.

L85: The first hypothesis, as written, is very general. Instead of using the term “fluctuations”, can you say there is enough evidence in the literature to be able to hypothesize that one can expect to see an increase in theta and decrease in beta, and why?

Methods:

L132: I think you meant to say “tandem stance” and not “Romberg stance”? Was there a specific reason to do all trials with right foot forward? (were all participants right foot dominant? What are the implications of it in EEG responses

There was substantially more EMG on the left side during pull perturbations while standing - so it would be expected somewhat higher cortical activity on right sensorimotor area for these perturbations, but it does not look like that was the case. I think this needs to be discussed more. Perhaps some sort of EEG-EMG coherence analyses may help shed more light on the origins of the asymmetrical muscle but not cortical response during pull perturbations while standing with left foot at the back during tandem stance (MAJOR)

L144: “the recording”

L159: I wonder why the authors chose to do a simple factorial ANOVA and not a repeated measure factorial ANOVA for this repeated design? (MAJOR)

L161: “Bonferroni”, also why Bonferroni was used for some and FDR for other tests? What was the corrected alpha? Or does the software adjust the p-value so you can keep alpha as 0.05?

L174: “the half-second”

L197: Spell out standard deviation at first use

L200: Please justify the use of 20 SDs used for ASR? Given the nature of the study (perturbations), the risk of motion artifacts cannot be overemphasized. Can the authors SHOW that the cleaned EEG data were free of motion artifacts? Please see recent publications from Ferris et al group (MAJOR)

L212: Perhaps some reference for k value will be good here.

L236: “MATLAB” Also mention the version, Mathworks, Natick, MA

L252: Please include reference here to justify the choice of synchronization band (4-13), or explain.

Results/Figures:

Fig. 1: L818: “visual rotation”

Fig. 7: L891: “significant increases”; L892: “increase”

Fig. 10: L918: Remove “indicates”; What does the error bar indicate in this figure?

The onset latency of Left SM and SMA seem really short (and possibly significantly different from that of right SM) during stand pull (red bars) in the upper panel. Could this be related to higher EMG on left leg for this task? These results require further explanation (MAJOR).

Fig. 11: L933 Remove the extra “and”

Discussion

L433: Unclear what is the purpose of adding the Varghese et al 2017 reference if yours is the first study for.....?

The authors should consider removing the claim that their study is the “first analyzed EEG time-frequency during balance perturbations in source space “ as this is not exactly the case nor the main msg of the study.

L464: “back leg was...stabilization in our paradigm”

It would be useful to add the relevant figure numbers in the discussion section to aid the reader

L524: “young adults EEG”

L525: “especially in..”

L533: “Similar..”

L542: “similar to””
